# Synergistic Effects of Medium-Chain Triglyceride Supplementation and Resistance Training on Physical Function and Muscle Health in Post-Stroke Patients

**DOI:** 10.3390/nu17091599

**Published:** 2025-05-07

**Authors:** Yoshihiro Yoshimura, Fumihiko Nagano, Ayaka Matsumoto, Sayuri Shimazu, Ai Shiraishi, Yoshifumi Kido, Takahiro Bise, Takenori Hamada, Kouki Yoneda

**Affiliations:** Center for Sarcopenia and Malnutrition Research, Kumamoto Rehabilitation Hospital, Kumamoto 869-1106, Japan; akro1029@gmail.com (F.N.); ayk1224mtmt@gmail.com (A.M.); shimazu@kumareha.jp (S.S.); ai.shiraishi0913@gmail.com (A.S.); kidonii921@yahoo.co.jp (Y.K.); asian.dub.foundation00@gmail.com (T.B.); t-hamada@kumareha.jp (T.H.); koukhun@gmail.com (K.Y.)

**Keywords:** medium-chain triglycerides, chair-stand exercise, sarcopenia, convalescent rehabilitation, activities of daily living, muscle health

## Abstract

**Background/Objectives**: Sarcopenia and malnutrition are common in post-stroke patients, impairing recovery. Medium-chain triglycerides (MCT) may support muscle metabolism, while chair-stand exercises improve strength and mobility. However, their combined effects remain unclear. This study evaluated the synergistic effects of MCT supplementation and high-frequency chair-stand exercise on physical function and muscle health in post-stroke patients. **Methods**: A retrospective cohort study included 1080 post-stroke patients categorized into three groups: (1) MCT supplementation alone, (2) chair-stand exercise alone, and (3) both combined. MCT supplementation consisted of ~40 g/day MCT oil or powder. Functional outcomes were assessed using the Functional Independence Measure (FIM-motor), while muscle health was evaluated by handgrip strength (HGS) and skeletal muscle index (SMI). Multiple linear regression analyses were performed, adjusting for confounders. **Results**: The combined intervention group showed significantly greater improvements in FIM-motor scores at discharge (B = 8.79, 95% CI: 5.64–11.95, β = 0.32, *p <* 0.001) and FIM-motor gain (B = 6.02, 95% CI: 3.42–8.62, β = 0.29, *p <* 0.001) compared to the control. Increases in HGS (B = 2.441, 95% CI: 0.483–4.398, β = 0.18, *p* = 0.015) and SMI (B = 0.194, 95% CI: 0.102–0.419, β = 0.12, *p* = 0.039) were also observed. Chair-stand exercise was performed a median of 62 times/day and independently improved outcomes, while MCT alone had limited effects. **Conclusions**: MCT supplementation combined with chair-stand exercise enhances functional recovery and muscle health in post-stroke patients, supporting its role in rehabilitation. Further research is needed to evaluate long-term effects and to examine the pharmacokinetics of MCTs, including blood concentrations, in broader populations.

## 1. Introduction

Sarcopenia and malnutrition are prevalent conditions in older adults, and they are particularly common in post-stroke patients undergoing rehabilitation [1,2]. In community-dwelling older individuals, estimates of sarcopenia prevalence range from roughly 10% to 40% depending on diagnostic criteria [3,4,5]. Among stroke survivors, sarcopenia is even more frequent: a recent meta-analysis found that about 42% of post-stroke patients meet criteria for sarcopenia [6]. Likewise, malnutrition or risk of malnutrition is widespread after stroke, with reported frequencies between 6% and 62% in acute and rehabilitation settings [7,8,9,10]. These conditions often coexist and have serious clinical consequences [11,12,13,14,15]. Muscle wasting and undernutrition lead to declines in physical function—for example, reduced mobility and impairment in activities of daily living (ADL)—which can impede recovery and prolong disability [16,17,18]. Indeed, the presence of sarcopenia at the time of stroke has been associated with poorer rehabilitation outcomes [16]. Conversely, improvements in muscle mass and strength during recovery correspond to better functional status and autonomy [19,20,21,22]. For instance, stroke patients who showed resolution of sarcopenia during inpatient rehabilitation achieved higher Functional Independence Measure (FIM) scores and had a greater likelihood of home discharge than those who remained sarcopenic [23,24,25]. These observations underscore that combating malnutrition and sarcopenia to improve physical function could lead to better overall outcomes in elderly and post-stroke populations.

Nutritional supplementation has emerged as a potential strategy to counter sarcopenia [26,27]. In particular, medium-chain triglycerides (MCT) have drawn attention for their metabolic and muscle health benefits [26,28,29,30]. MCT are rapidly absorbed fatty acids that provide an efficient energy substrate, and recent evidence suggests MCT intake may help maintain muscle mass and improve functional performance [31,32,33]. For example, in frail older adults, three months of low-dose MCT supplementation (6 g/day of octanoic + decanoic acids) significantly increased muscle mass and strength compared to an isocaloric long-chain fat, indicating a tangible benefit for muscle health [32,34,35]. MCT supplementation has also been linked to improved exercise capacity. In one randomized trial, healthy older adults who received 6 g/day of MCT oil during a 12-week walking exercise program showed greater gains in knee extension and grip strength than those who exercised without MCT [32]. The authors noted that combining aerobic exercise with MCT appeared effective at preventing declines in muscle strength and promoting additional gains, potentially by enhancing energy metabolism in muscle [32]. These findings collectively highlight MCT’s potential to improve metabolic status, preserve lean mass, and support physical function in vulnerable older populations.

Exercise interventions are another cornerstone for improving muscle function in older adults [36]. Resistance training in particular has well-documented efficacy in enhancing strength and functional ability in older adults [37,38,39,40]. A recent meta-analysis of 21 trials (*n* = 1610) confirmed that progressive resistance exercise produces significant improvements in muscle strength (upper and lower body) and modest gains in physical functioning in adults over 60 [41]. Such training can translate into better mobility, balance, and capacity for ADL. Even relatively simple, low-intensity resistance exercises can be beneficial [42,43,44]. For instance, chair-stand exercise—a repetitive sit-to-stand movement performed in groups as part of rehab—has been shown to improve mobility and ADL performance in clinical populations [45,46,47]. In stroke patients, incorporating daily chair-stand sessions into standard therapy was reported to boost their ADL independence [45]. These examples illustrate that engaging older or post-stroke patients in appropriate resistance or functional exercise can counteract declines in muscle function and improve overall physical performance.

While nutritional supplementation (such as MCT) and exercise each can positively impact muscle health, their combined effects remain underexplored. A number of trials have examined exercise or dietary interventions separately in older adults with sarcopenia, but far fewer have addressed using them together [48]. Some combined approaches (mostly pairing resistance exercise with protein supplementation) suggest additive benefits for muscle mass and strength, but overall findings have been inconsistent [49,50,51]. Notably, evidence is lacking on the specific combination of MCT supplementation with resistance exercise. To our knowledge, no study to date has evaluated whether adding MCT—a targeted nutritional support—to an exercise regimen yields superior improvements in muscle health or physical ability compared to exercise or nutrition alone in post-stroke patients. This gap in the literature is important, as an integrated intervention could synergistically address both nutritional and functional deficits in recovering stroke survivors.

Therefore, the present study aimed to investigate the combined effect of MCT supplementation and resistance exercise on physical function and muscle health in post-stroke inpatients undergoing rehabilitation. We conducted a retrospective cohort study in a convalescent rehabilitation hospital focusing on stroke survivors with reduced physical function. We compared muscle health indicators (e.g., muscle mass, handgrip strength) and functional outcomes (e.g., ADL independence) between those who received the MCT + exercise intervention and those who did not. We hypothesized that the combination of MCT intake and chair-stand exercise would lead to greater improvements in muscle mass, strength, and physical function during the rehabilitation stay relative to usual care alone.

## 2. Materials and Methods

### 2.1. Participants and Setting

A single-center, retrospective cohort study was conducted at a rehabilitation hospital with 135-bed convalescent rehabilitation wards. The study population included all stroke patients with cognitive decline who were consecutively admitted and discharged from 2016 to 2023. Exclusion criteria were patients with altered consciousness on admission, clinically significant limb edema or abnormal fluid balance, incomplete data, and patients who refused consent. Patients were observed until discharge.

The convalescent rehabilitation regimen, which was conducted during hospitalization for up to three hours daily, was managed by a multidisciplinary group of medical professionals and customized to suit each patient’s specific functional abilities and constraints [52]. This holistic approach integrated physical, occupational, speech, and hearing therapies, along with dietary assistance [53], oral hygiene and rehabilitation [54], and pharmaceutical oversight [55]. The physical therapy aspect encompassed treatments focusing on affected limbs, enhancing joint mobility, walking instruction, full-body exercises, and training for everyday tasks [56].

### 2.2. Data Collection

Data on patient demographic and clinical characteristics were systematically collected upon admission. Recorded characteristics included age, sex, stroke type, history of stroke, and days from stroke onset to admission. Nutritional status was screened on the admission day using the Mini Nutritional Assessment—Short Form (MNA-SF) [57].

Physical and cognitive functions were evaluated using the FIM, including both motor and cognition subdomains [58]. Lower limb motor function severity was assessed by the (BRS-lower extremity) [59]. Pre-stroke functional status was assessed using the modified Rankin Scale (mRS) [60], while comorbidities were quantified by Charlson’s Comorbidity Index (CCI) [61]. All assessment tools, including the MNA-SF, FIM, BRS, mRS, and CCI, were administered in their validated Japanese versions. These instruments are widely used in Japanese clinical and research settings, with established reliability and validity.

Skeletal muscle mass was assessed using multi-frequency bioelectrical impedance analysis (BIA; InBody S10, InBody, Tokyo, Japan), and skeletal muscle index (SMI) was calculated as appendicular muscle mass divided by height squared (kg/m^2^) [62]. The device estimates muscle mass using direct segmental impedance values across multiple frequencies without applying age- or sex-dependent prediction equations [63]. In this study, SMI was analyzed as a continuous variable and not categorized using diagnostic cutoff values. BIA has been validated in older adults and post-stroke patients with hemiparesis, showing good agreement with DXA and acceptable accuracy even in neurologically impaired older populations [64,65]. Measurements were performed in the supine position within three days of admission and discharge, using bilateral limb electrodes to obtain whole-body impedance. Patients with visible edema were excluded, and those with mild edema were screened using extracellular water (ECW) ratio and intracellular-to-extracellular water (ICW/ECW) ratio; individuals with abnormal fluid distribution were also excluded to reduce measurement bias [66]. Handgrip strength was measured three times using a Smedley hand dynamometer (TTM, Tokyo, Japan) on the non-dominant or unaffected hand, and the highest value was recorded for analysis [67]. Nutritional intake (energy and protein) was evaluated based on patients’ average weekly consumption normalized by actual body weight [68].

Daily rehabilitation therapy administered during hospitalization was recorded as the cumulative units provided, with one unit representing a 20-min therapy session [69]. The number of oral medications prescribed at admission and the length of hospitalization were recorded in the patient’s file.

### 2.3. MCT Supplementation

The provision of MCT-enhanced rice to patients was integrated as part of their routine clinical care regimen, being administered three times daily. At the hospital where the study was conducted, the implementation of MCT-enhanced rice was adopted as one of the nutritional support options for patients suffering from malnutrition. The decision to provide MCT-enhanced rice was made through multidisciplinary nutritional conferences involving the patient’s attending physicians, registered dietitians, and other members of the nutrition support team, who carefully evaluated the patient’s nutritional needs and determined that MCT supplementation would be appropriate.

Overall, patients meeting the following criteria were provided with MCT-enhanced rice as part of their nutritional regimen:

- Malnutrition assessed as MNA-SF ≤ 7;
- Low body weight with BMI < 18.5 kg/m^2^;
- Low skeletal muscle mass assessed via bioelectrical impedance analysis (BIA) (men: SMI < 7.0 kg/m^2^, women: SMI < 5.7 kg/m^2^);
- Dysphagia or swallowing difficulty with FILS ≤ 7;
- Patients for whom weight loss would be a concern under regular nutritional management.

The MCT-enhanced rice was prepared as follows:

1. Ingredients: 150 g soft rice (equivalent to 100 g regular rice), 12 g 100% pure MCT oil, and 1.5 g 100% pure MCT powder (Nisshin OilliO Group, Ltd., Tokyo, Japan).
2. The MCT oil and powder were measured, mixed, and combined with the soft rice.
3. The formulation was determined through experimentation to optimize taste, texture, appearance, and odor.
4. Soft rice was chosen for ease of mixing with the powder and oil.

Nutritional composition of the MCT-enhanced rice was as follows:

1. One cup of MCT-enhanced rice (exposure of interest in this study) contains:
  - 305 kcal of energy;
  - 2.5 g of protein;
  - 13.4 g of fat, including 11.8 g of MCT.
2. In contrast, one cup of regular rice (control in this study) contains:
  - 168 kcal of energy;
  - 2.5 g of protein;
  - 0.3 g of fat.

In addition to the MCT-supplemented rice, all patients received standard hospital meals three times daily, planned and adjusted by registered dietitians according to individual nutritional status, swallowing function, and comorbidities. The meals typically included soft rice or porridge, soup, protein sources (fish, chicken, tofu, or eggs), and cooked vegetables. The planned macronutrient distribution of the total diet was approximately 55–60% carbohydrates, 15–20% protein, and 20–25% fat. Importantly, MCTs were not present in the standard hospital diet and were provided only through the MCT-supplemented rice, making this the sole source of medium-chain triglyceride intake in the intervention. Daily energy and protein intake during hospitalization were assessed using average weekly food intake records normalized to body weight.

### 2.4. Chair-Stand Exercise

In addition to the standard rehabilitation program, patients participated in chair-stand exercises as a form of full-body resistance training [45,46,47]. These exercises were conducted using a standard chair, a platform, or a wheelchair, with the seat height adjusted to accommodate each patient’s physique, typically ranging from 40 to 50 cm. Parallel bars and handrails were available for support when needed, and rehabilitation therapists provided assistance to those unable to stand independently.

Each session lasted 20 min, during which patients performed repeated sit-to-stand movements at a controlled pace of approximately one repetition every 8 s, with a maximum of 120 repetitions per session. On the initial day, patients performed a limited number of repetitions, which was progressively increased as muscle strength and endurance improved. The exercise intensity was tailored to each individual’s functional status, with the rehabilitation therapist adjusting the number of repetitions based on symptom severity, patient motivation, pain levels, and need for assistance. Generally, the exercise was performed at 20–30% of maximal repetitions, making it a slow and controlled regimen suitable for older adults with reduced physical capacity.

Chair-stand exercises were conducted twice daily as part of a group-based rehabilitation program. The frequency and rate of progression varied among patients, depending on their endurance and physical condition. This structured exercise regimen was considered a safe and effective method for enhancing muscle mass and strength in older adults undergoing rehabilitation.

### 2.5. Outcomes

The primary outcome of this study was the motor subscale of the FIM (FIM-motor), which evaluates ADL [58]. The primary endpoints included the FIM-motor score at discharge and its gain, defined as the change in FIM-motor from admission to discharge. The FIM is a standardized, clinician-reported tool consisting of 18 items that assess functional abilities across multiple domains, including self-care, continence, mobility, transfers, communication, and cognition. Each item is rated on a 7-point scale, with higher scores indicating greater independence. The total FIM score ranges from 18 to 126, and it is subdivided into motor and cognitive components. The FIM-motor subscale specifically ranges from 13 to 91. This instrument has demonstrated strong reliability and validity, with high interrater reliability for total, motor, and cognitive scores.

Secondary outcomes included handgrip strength (HGS) and SMI at discharge. HGS was measured three times using a Smedley hand dynamometer on the non-dominant hand, or the unaffected hand in cases of hemiparesis, with the highest value recorded [70]. If a patient was unable to complete the assessment, HGS was documented as 0.0 kg. SMI was assessed via BIA using a validated multi-frequency BIA device [71]. It was calculated by dividing the measured skeletal muscle mass by height squared (kg/m^2^). SMI measurements were conducted within three days of both admission and discharge to capture relevant changes during the rehabilitation period.

To minimize measurement bias, rehabilitation therapists and nurses responsible for FIM and HGS assessments were independent from the personnel managing data collection, statistical analyses, and interpretation of study findings. This procedural separation ensured the objectivity and integrity of outcome measurements, reinforcing the reliability of the study results.

### 2.6. Sample Size Calculation

The sample size was calculated using data from our previous study [72], the results of which showed that the FIM-motor of patients admitted to the hospital was normally distributed with a standard deviation (SD) of 26.0. Assuming a true mean difference of 17 between patients with and without sarcopenia and impaired oral health [53], a sample size of at least 97 participants in each of the two groups was required to reject the null hypothesis with 80% power at an alpha level of 0.05.

### 2.7. Statistical Analysis

This study was designed as a retrospective cohort analysis rather than a randomized controlled trial, which precludes establishing a definitive causal relationship. To enhance the validity of causal inferences, potential confounders were carefully selected and adjusted for in multivariate analyses. The study investigated the impact of MCT supplementation and/or high-frequency chair-stand exercise on patient outcomes by classifying cases into three exposure patterns: (1) MCT supplementation only, (2) high-frequency chair-stand exercise only, and (3) both interventions combined.

For continuous variables, parametric data were reported as means and standard deviations (SD), while non-parametric data were expressed as medians and interquartile ranges (IQR). Normality was assessed using the Shapiro–Wilk test and confirmed with the Kolmogorov–Smirnov test, with non-parametric classification assigned to variables showing *p <* 0.05 in either test or to ordinal variables. Ordinal variables, such as the BRS for the lower extremity and the pre-stroke mRS, were analyzed accordingly. Categorical variables were expressed as frequencies and percentages (%). Baseline characteristics between groups were compared using the *t*-test for parametric data, the Mann–Whitney U test for non-parametric data, and the chi-square test for categorical variables [73].

To assess the associations between MCT supplementation, chair-stand exercise, and study outcomes—including FIM-motor score at discharge, FIM-motor gain, discharge HGS, and discharge SMI—multiple linear regression analyses were conducted. Confounders were determined based on clinical relevance and prior literature and included age, sex, baseline values of outcomes (FIM-motor, SMI, and HGS), stroke type, pre-stroke mRS, CCI, BRS-lower extremity, FIM-cognition, protein intake, number of medications, MNA-SF, and HGS at admission [74,75,76,77,78]. Multicollinearity was evaluated using the variance inflation factor (VIF), where a VIF value between 1 and 10 was considered acceptable [79]. A *p*-value of <0.05 was deemed statistically significant. All statistical analyses were performed using IBM SPSS version 21 (Armonk, NY, USA).

### 2.8. Ethics

This study was approved by the Institutional Review Board of the hospital where the research was conducted (approval ID: 35-250313, approved on 13 March 2025). Given the retrospective study design, obtaining written informed consent from participants was not feasible. However, an opt-out procedure was implemented, allowing participants the opportunity to withdraw from the study at any time. This study was conducted in accordance with the ethical principles of the 1964 Declaration of Helsinki and its subsequent revisions, as well as the Ethical Guidelines for Medical and Health Research Involving Human Subjects (Provisional Translation, March 2015).

## 3. Results

**Figure 1:**
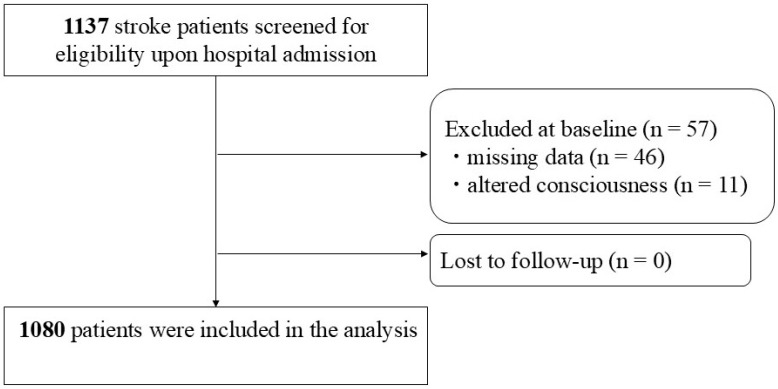
Flowchart of participant screening, inclusion criteria, and follow-up.

Over the study period, 1137 stroke patients were newly admitted to the wards. Of these, 46 patients with incomplete data and 11 with altered consciousness were excluded from the analysis. Following the application of exclusion criteria, a total of 1080 patients were included in the final analysis (Figure 1).

The baseline characteristics of the 1080 post-stroke rehabilitation patients are presented in Table 1. The median age was 75.6 years, and 54.1% of the cohort was male. The most common stroke type was cerebral infarction (63.6%), followed by cerebral hemorrhage (29.7%) and subarachnoid hemorrhage (6.7%). The median duration from stroke onset to hospital admission was 14 days. Patients exhibited moderate to severe impairment in activities of daily living (ADL) at admission, with a median FIM-motor score of 46 and a total FIM score of 66. Among nutritional and muscle strength indicators, the median HGS was 18.8 kg, the SMI was 6.3 kg/m^2^, and the MNA-SF score was 7. The median body mass index (BMI) was 22.3 kg/m^2^. Comparison of patients receiving MCT-enhanced rice and/or high-frequency chair-stand exercise showed significant differences across several variables. Patients who received only MCT (MCT+/EXERCISE−) were significantly older (78.9 vs. 75.6 years, *p <* 0.001) and had lower baseline FIM-motor scores (15 vs. 46, *p <* 0.001). Similarly, patients receiving only high-frequency chair-stand exercise (MCT−/EXERCISE+) exhibited significantly higher HGS (23.5 vs. 18.8 kg, *p <* 0.001) and SMI (6.6 vs. 6.3 kg/m^2^, *p <* 0.001). No significant differences were observed in protein intake or the number of prescribed medications at admission.

Table 2 presents the results of the multiple linear regression analyses examining the associations between MCT supplementation and/or high-frequency chair-stand exercise with FIM-motor score at discharge and FIM-motor gain. Patients who received MCT supplementation alone (MCT+/EXERCISE−) exhibited significantly lower FIM-motor scores at discharge (β = −4.82, 95% CI: −7.36 to −2.29, *p <* 0.001) and lower FIM-motor gain (β = −2.94, 95% CI: −5.21 to −0.67, *p* = 0.011) compared to the reference group (MCT−/EXERCISE−). In contrast, those who participated in high-frequency chair-stand exercise alone (MCT−/EXERCISE+) showed significantly greater improvements in FIM-motor score at discharge (β = 6.31, 95% CI: 3.89 to 8.72, *p <* 0.001) and FIM-motor gain (β = 4.82, 95% CI: 2.45 to 7.18, *p <* 0.001). Notably, patients who received both MCT supplementation and high-frequency chair-stand exercise (MCT+/EXERCISE+) demonstrated the highest functional gains, with a significantly greater FIM-motor score at discharge (β = 8.79, 95% CI: 5.64 to 11.95, *p <* 0.001) and FIM-motor gain (β = 6.02, 95% CI: 3.42 to 8.62, *p <* 0.001) compared to the reference group. The VIF confirmed no significant multicollinearity among the included variables. These findings suggest that high-frequency chair-stand exercise independently contributes to better functional recovery, while the combined intervention of MCT supplementation and exercise may provide an additive benefit.

Table 3 presents the results of the multiple linear regression analyses examining the associations between MCT supplementation and/or high-frequency chair-stand exercise with HGS and SMI at discharge. Patients who received MCT supplementation alone (MCT+/EXERCISE−) showed no significant improvement in HGS (β = 0.396, 95% CI: −1.059 to 1.851, *p* = 0.593) or SMI (β = −0.259, 95% CI: −0.515 to 0.053, *p* = 0.147) compared to the reference group (MCT−/EXERCISE−). Conversely, patients who engaged in high-frequency chair-stand exercise alone (MCT−/EXERCISE+) exhibited significantly greater HGS at discharge (β = 1.308, 95% CI: 0.255 to 2.361, *p* = 0.015) and higher SMI (β = 0.146, 95% CI: 0.030 to 0.323, *p* = 0.034). Notably, patients receiving both MCT supplementation and high-frequency chair-stand exercise (MCT+/EXERCISE+) showed the greatest improvements in muscle health, with significantly higher HGS at discharge (β = 2.441, 95% CI: 0.483 to 4.398, *p* = 0.015) and increased SMI (β = 0.194, 95% CI: 0.102 to 0.419, *p* = 0.039) compared to the reference group. The VIF analysis confirmed no significant multicollinearity among the variables. These results suggest that high-frequency chair-stand exercise contributes to improved muscle strength and mass, with the combination of MCT supplementation and exercise showing an additive effect.

## 4. Discussion

This retrospective cohort study investigated the synergistic effects of MCT supplementation and high-frequency chair-stand exercise on functional recovery and muscle health in 1080 post-stroke patients. The primary findings demonstrated that combining MCT-enhanced rice with high-frequency chair-stand exercise resulted in significantly greater improvements in ADL and muscle health compared to either intervention alone or usual care. Notably, high-frequency chair-stand exercise independently drove functional gains, while MCT supplementation amplified these benefits when combined with exercise, suggesting a complementary mechanism of action.

MCT supplementation combined with structured resistance training was associated with accelerated ADL recovery during post-stroke rehabilitation, highlighting a novel synergistic strategy with substantial clinical relevance for nutritional support in rehabilitative care. Patients who received both interventions demonstrated significantly greater functional gains compared to those receiving resistance training alone, with an adjusted mean FIM-motor improvement of nearly 10 points—an absolute difference considered clinically meaningful in post-stroke rehabilitation. This finding emphasizes MCT’s potential to enhance exercise-induced neuromuscular remodeling. From a metabolic perspective, MCT-derived ketones serve as a readily available energy source, supporting the preservation of lean muscle mass during rehabilitation-induced caloric deficits and facilitating oxidative metabolism within skeletal muscle [27,80,81]. Notably, these benefits were observed only when MCT supplementation was combined with structured exercise, as isolated MCT use in sedentary patients was paradoxically associated with poorer outcomes, underscoring the necessity of integrating targeted nutritional strategies with mechanical loading [82].

MCT supplementation in conjunction with structured resistance training fosters significant improvements in muscle health, offering a promising strategy for mitigating sarcopenia in these patients. This study demonstrated that the combined intervention led to greater skeletal muscle retention and enhanced strength recovery compared to resistance training alone, reinforcing the importance of integrating nutritional and mechanical stimuli for optimal rehabilitation outcomes. From a metabolic perspective, MCT-derived ketones may serve as an efficient energy substrate that supports prolonged muscular activity while modulating anabolic pathways, such as mTOR, to counteract muscle catabolism [83,84]. Although the exact mechanisms were not directly assessed in our study, it is plausible that structured resistance training stimulated muscle protein synthesis, while MCT supplementation supported energy metabolism during exercise-induced caloric deficits. Clinically, these findings suggest that a dual approach combining MCT supplementation with targeted resistance training may serve as an effective countermeasure against sarcopenia, ultimately promoting greater functional independence in stroke survivors. Future research should explore individualized MCT dosing strategies and investigate the long-term effects of this integrated intervention to optimize muscle health recovery in rehabilitative settings.

A key strength of this study is its large sample size, which enhances statistical power and generalizability to post-stroke rehabilitation populations. Additionally, the study utilizes real-world clinical data, providing valuable insights into the synergistic effects of MCT supplementation and structured resistance training in a practical rehabilitation setting. Importantly, high-protein nutritional support was standardized across all patients, allowing for a more precise evaluation of MCT’s independent effect on muscle mass augmentation. The use of well-validated outcome measures, including FIM-motor, handgrip strength, and skeletal muscle index, further strengthens the reliability of the findings.

Several limitations should be acknowledged. The retrospective, non-randomized design inherently limits causal inferences, and potential residual confounding cannot be entirely excluded despite rigorous statistical adjustments. Baseline differences between groups and the absence of a placebo intervention in the non-MCT groups may also have introduced bias. Given the strong correlation between energy and protein intake, only protein intake was adjusted as a confounding factor in the multivariate analysis. While this approach allowed for an independent assessment of MCT’s contribution to muscle mass gains, total energy intake was not strictly controlled, which may have influenced outcomes. Additionally, the long-term effects of the intervention remain unclear, warranting further prospective studies to assess sustained benefits and optimize implementation strategies. Moreover, no blood sampling was conducted to assess plasma MCT or triglyceride levels, limiting our ability to verify the metabolic response. Based on previous literature, oral MCT administration at similar doses typically results in transient increases in plasma medium-chain fatty acid concentrations [69], but this could not be confirmed in our study. Lastly, although the median protein intake in this study was approximately 1.0 g/kg/day, which is lower than the 1.2–1.5 g/kg/day recommended for older adults undergoing rehabilitation, this level reflects actual dietary intake in many clinical settings. Future studies should refine intervention protocols, explore long-term outcomes, and employ randomized, placebo-controlled designs to establish more robust evidence for rehabilitation strategies [85,86].

## 5. Conclusions

This study highlights the synergistic benefits of MCT supplementation and structured resistance training in post-stroke rehabilitation, demonstrating significant improvements in ADL recovery and muscle health. Importantly, these benefits were observed independently of overall protein intake, a factor well known to influence muscle health and ADL outcomes. This suggests that MCT supplementation may provide additional metabolic support for exercise beyond the effects of dietary protein optimization. These findings support the integration of targeted nutritional strategies alongside exercise-based interventions to optimize rehabilitative outcomes. Future prospective studies are warranted to validate these results and further refine intervention protocols.

## Figures and Tables

**Table 1:** Baseline characteristics of post-stroke rehabilitation patients stratified by MCT provision and/or high-frequency chair-stand exercise.

Variable	Total N = 1080	Patients Receiving Either MCT ^1^ or EXERCISE ^2^, or Both			
		MCT+/EXERCISE− N = 126	MCT−/EXERCISE+ N = 468	MCT+/EXERCISE+ N = 58	*p*-Value ^#^
Age, *years*	75.6 (9.3)	78.9 (11.2)	69.1 (13.9)	71.0 (14.0)	<0.001
Sex, *male*	584 (54.1)	56 (44.4)	283 (60.5)	39 (67.2)	<0.001
Stroke type					
Cerebral infarction	687 (63.6)	79 (62.7)	302 (64.5)	29 (50.0)	0.109
Cerebral hemorrhage	321 (29.7)	43 (34.1)	127 (27.1)	24 (41.4)	0.083
SAH	72 (6.7)	4 (3.2)	39 (8.3)	5 (8.6)	0.210
Onset-to-admission time, *days*	14 [11, 22]	17 [12, 24]	14 [11, 21]	15 [10, 27]	0.031
BRS, lower extremity	5 [3, 6]	4 [2, 5]	5 [4, 6]	4 [2, 6]	0.171
Stroke history	263 (24.4)	39 (31.0)	99 (21.2)	16 (27.6)	0.045
Pre-stroke mRS, *score*	0 [0, 2]	1 [0, 3]	0 [0, 1]	0 [0, 1]	0.201
CCI, *score*	3 [2, 4]	3 [3, 4]	3 [1, 3]	3 [2, 4]	0.146
FIM, *score*					
Total	66 [34, 92]	29 [22, 45]	82 [61, 103]	36 [27, 64]	<0.001
Motor	46 [20, 67]	15 [13, 28]	58 [40, 75]	21 [14, 42]	<0.001
Cognition	21 [12, 27]	12 [7, 18]	25 [19, 30]	17 [10, 23]	<0.001
MNA-SF, *score*	7 [5, 9]	5 [3, 6]	8 [6, 10]	5 [3, 7]	<0.001
HGS, kg	18.8 [10.0, 27.5]	8.5 [0.0, 17.7]	23.5 [16.7, 31.9]	16.3 [8.3, 22.8]	<0.001
BMI, kg/m^2^	22.3 [19.8, 24.7]	20.1 [18.1, 21.8]	23.0 [20.7, 25.4]	21.6 [19.4, 24.1]	<0.001
SMI, kg/m^2^	6.3 [5.3, 7.3]	5.3 [4.4, 6.4]	6.6 [5.7, 7.5]	6.3 [5.4, 7.2]	<0.001
Nutrition intake					
Energy, kcal/kg/day	27.3 [23.3, 31.8]	27.10 [24.10, 32.03]	26.9 [23.1, 30.7]	24.7 [22.6, 28.8]	0.061
Protein, g/kg/day	1.0 [0.9, 1.2]	1.0 [0.9, 1.2]	1.0 [0.9, 1.2]	1.0 [0.8, 1.1]	0.101
Length of hospital stay, *days*	86 [53, 128]	122 [88, 149]	72 [46, 115]	138 [84, 154]	<0.001
Number of medications, *number*	5 [3, 7]	6 [4, 8]	5 [3, 7]	5 [3, 7]	0.056
MCT ^1^, *n (%)*	184 (17.0)	-	-	-	-
Chair-stand exercise, *frequency*	62 [36, 96]	32 [17, 46]	97 [78, 124]	89 [74, 109]	-
Rehabilitation therapy ^3^, *units*	8.2 [7.5, 8.6]	8 [6, 8]	8 [8, 8]	8 [7, 8]	0.164

Parametric values are presented as mean (SD); nonparametric values as median [interquartile range]; frequency as n (%).^1^ MCT: Provision of MCT-enhanced rice. ^2^ EXERCISE: Chair-stand exercise. ^3^ Rehabilitation therapy (including physical, occupational, and speech and swallowing therapy) performed during hospitalization (1 unit = 20 min). ^#^ Kruskal–Wallis test. Abbreviations: BMI, body mass index; BRS, Brunnstrom Recovery Stage; CCI, Charlson’s Comorbidity Index; FIM, Functional Independence Measure; HGS, handgrip strength; mRS, modified Rankin scale; SAH, subarachnoid hemorrhage; SMI, skeletal muscle mass index.

**Table 2:** Multiple linear regression analysis examining the association between MCT provision and/or high-frequency chair-stand exercise with FIM-motor at discharge and its gain.

	FIM-Motor at Discharge			FIM-Motor Gain		
	B (95% CI)	b	*p*-Value	B (95% CI)	b	*p*-Value
MCT ^1^: Yes EXERCISE ^2^: No	−1.33 (−4.86, 2.19)	−0.018	0.458	−1.33 (−4.86, 2.19)	−0.028	0.458
MCT ^1^: No EXERCISE ^2^: Yes	5.37 (2.83, 7.90)	0.103	<0.001	5.37 (2.83, 7.90)	0.132	<0.001
MCT ^1^: Yes EXERCISE ^2^: Yes	9.80 (5.09, 14.51)	0.193	<0.001	9.80 (5.09, 14.52)	0.197	<0.001

All multivariate analyses were adjusted for the following admission covariates: age, sex (male), FIM-motor, FIM-cognition, stroke type, pre-stroke mRS, CCI, BRS-lower extremity, protein intake, HGS, MNS-SF, and number of drug prescriptions. ^1^ MCT: Provision of MCT-enhanced rice. ^2^ EXERCISE: Chair-stand exercise. BRS: Brunnstrom Recovery Stage, CCI: Charlson’s Comorbidity Index, FIM: Functional Independence Measure, HGS: handgrip strength, mRS: modified Rankin scale

**Table 3:** Multiple linear regression analysis examining the association between MCT provision and/or high-frequency chair-stand exercise and handgrip strength and skeletal muscle index at discharge.

	HGS at Discharge			SMI Discharge		
	B (95% CI)	b	*p*-Value	B (95% CI)	b	*p*-Value
MCT ^1^: Yes EXERCISE ^2^: No	0.39 (−1.05, 1.85)	0.012	0.593	−0.25 (−0.51, 0.05)	−0.063	0.147
MCT ^1^: No EXERCISE ^2^: Yes	1.30 (0.25, 2.36)	0.058	0.015	0.14 (0.03, 0.32)	0.043	0.34
MCT ^1^: Yes EXERCISE ^2^: Yes	2.44 (0.48, 4.39)	0.083	0.015	0.19 (0.10, 0.41)	0.084	0.039

All multivariate analyses were adjusted for the following admission covariates: age, sex (male), FIM-motor, FIM-cognition, stroke type, pre-stroke mRS, CCI, BRS-lower extremity, protein intake, HGS, MNS-SF, and number of drug prescriptions. ^1^ MCT: Provision of MCT-enhanced rice. ^2^ EXERCISE: Chair-stand exercise. BRS: Brunnstrom Recovery Stage, CCI: Charlson’s Comorbidity Index, FIM: Functional Independence Measure, HGS: handgrip strength, mRS: modified Rankin scale, SMI: skeletal muscle mass index.

## Data Availability

The data presented in this study are available on request from the corresponding author due to opt-out restrictions.
